# Graph-theoretical comparison of normal and tumor networks in identifying BRCA genes

**DOI:** 10.1186/s12918-017-0495-0

**Published:** 2017-11-22

**Authors:** Joaquin Dopazo, Cesim Erten

**Affiliations:** 1 Clinical Bioinformatics Research Area, Fundación Progreso y Salud, Hospital Virgen del Rocío, Sevilla, Spain; 2 Computer Engineering, Antalya Bilim University, Antalya, Turkey

**Keywords:** BRCA, Interactome, Network centrality

## Abstract

**Background:**

Identification of driver genes related to certain types of cancer is an important research topic. Several systems biology approaches have been suggested, in particular for the identification of breast cancer (BRCA) related genes. Such approaches usually rely on differential gene expression and/or mutational landscape data. In some cases interaction network data is also integrated to identify cancer-related modules computationally.

**Results:**

We provide a framework for the comparative graph-theoretical analysis of networks integrating the relevant gene expression, mutations, and potein-protein interaction network data. The comparisons involve a graph-theoretical analysis of normal and tumor network pairs across all instances of a given set of breast cancer samples. The network measures under consideration are based on appropriate formulations of various centrality measures: betweenness, clustering coefficients, degree centrality, random walk distances, graph-theoretical distances, and Jaccard index centrality.

**Conclusions:**

Among all the studied centrality-based graph-theoretical properties, we show that a betweenness-based measure differentiates BRCA genes across all normal versus tumor network pairs, than the rest of the popular centrality-based measures. The AUROC and AUPR values of the gene lists ordered with respect to the measures under study as compared to NCBI BioSystems pathway and the COSMIC database of cancer genes are the largest with the betweenness-based differentiation, followed by the measure based on degree centrality. In order to test the robustness of the suggested measures in prioritizing cancer genes, we further tested the two most promising measures, those based on betweenness and degree centralities, on randomly rewired networks. We show that both measures are quite resilient to noise in the input interaction network. We also compared the same measures against a state-of-the-art alternative disease gene prioritization method, MUFFFINN. We show that both our graph-theoretical measures outperform MUFFINN prioritizations in terms of ROC and precions/recall analysis. Finally, we filter the ordered list of the best measure, the betweenness-based differentiation, via a maximum-weight independent set formulation and investigate the top 50 genes in regards to literature verification. We show that almost all genes in the list are verified by the breast cancer literature and three genes are presented as novel genes that may potentialy be BRCA-related but missing in literature.

## Background

Cancer genes are involved in the dysfunction of a wide range of cellular functions including cell proliferation, angiogenesis, tumor invasion, DNA repair, chromosome stability, cell–cell communication, cell–matrix interactions, motility, metastasis, and apoptosis [1]. Much of recent cancer research has been devoted to identifying genes related to cancer initiation and progression computationally, and many different types of approaches have been suggested to this end. A comprehensive recent survey on computational approaches for the identification of cancer genes and pathways has been provided in [2].

One possible categorization of the computational approaches for cancer gene identification is based on the data they employ. Those employing mutations data to extract candidate cancer genes are based on the presupposition that driver genes can be identified via a thorough examination of recurrent mutations, whose observed frequency in a large cohort of cancer patients is much higher than expected. However usually a significantly low overlap in alterations of the alternative driver genes is observed, giving rise to what is known as *mutual exclusivity*. Several approaches relying on mutations data thus have developed specialized techniques to deal with the issue of exclusivity [3–7]. A second class of approaches consist of those employing gene expression data in the form of expression profiling, gene coexpression, or differential expression analysis [1, 8–10].

Recent integrative approaches employ one or both types of expression and mutations data together with interactions network data in the form of genetic or protein-protein interactions (PPI) [11–14]. Approaches combining gene expression data with the relevant interactions data in the context of long non-coding RNAs (lncRNA) have shown promising results in identfying lncRNA-disease associations [15–19]. Particularly, the interactome has demonstrated its usefulness in explaining the observed patterns of mutations either in healthy or in diseased individuals [20]. Rather than identifying a set of cancer-related genes, the goal of the integrative computational approaches usually is to extract modules deemed central to the cancer. HotNet2 employs a random-walk on the PPI network distributing the mutation frequencies of genes throughout the network, giving rise to a directed graph where the strongly connected components represent the output modules [21]. MEMCover combines mutual exclusivity data of mutations across several tissue types with the PPI network data to produce modules of cancer genes [22]. Although potentially useful for pan-cancer analysis, such approaches have limited use for specific cancer types where relatively small number of samples does not provide adequate information in the form of mutual exclusivity of the mutations. Furthermore they focus on the discovery of cancer modules rather than prioritizing individual genes as cancer drivers. By contrast, a recent cancer gene prioritization method, MUFFINN, applies a network-centric analysis of mutation data thereby integrating mutational information for individual genes and their neighbors in functional/interaction networks. It is suggested that MUFFINN’s cancer gene prioritization has good performance even in the setting where only data from a limited number of samples is employed [23].

We employ mutations data, gene expression data, as well as network data in the form of PPI networks, to identify individual driver genes related to breast cancer. The general framework consists of a comparative analysis of graph-theoretical measures. It is based on differential identification of breast cancer genes via a pairwise comparison of the values attained for a specific graph-theoretical measure applied on a normal and a tumor tissue sample over all available samples. Although recent studies comparing normal and tumor samples with regards to changes in genetic data including those in the form of mRNA expression, miRNA expression, or methylation alterations have beeen suggested, our study extends these approaches by introducing a network aspect and several common graph centrality measures, into the comparison [24–26]. We note that graph centralities have been employed in the context of identifying breast cancer genes in the past [27]. Such an approach has been revisited recently and an extension employing two different machine learning classifiers on computed centrality scores have been suggested [28]. However rather than incorporating gene expression and mutations data, as is done in our study, these approaches are limited to *gene signatures*; a set of centrality measures have been applied to PPI networks limited to genes already known to be related to breast cancer, to assign a degree of importance. Furthermore, our framework involving a comparative analysis of network centralities in pairs of graphs generated from normal and tumor tissue samples introduces a novelty that enables a differential analysis of genes involved in breast cancer.

## Methods

**Fig. 1:**
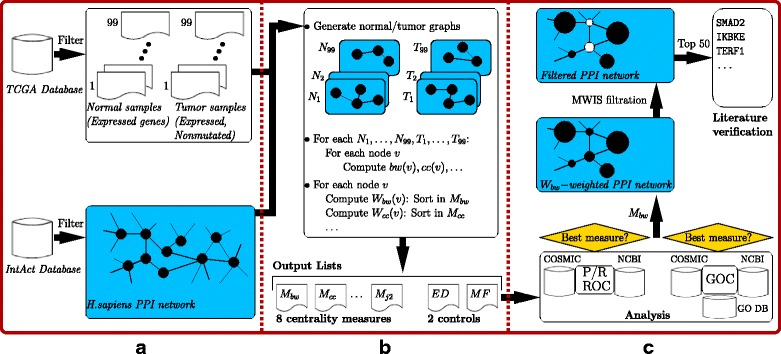
Flowchart summarizing the overall methodology. Flowchart summarizing the overall methodology. The first step depicted in part-a consists of data processing and necessary filtrations of the input databases TCGA and IntAct. The second step depicted in part-b involves generation of pairs of normal/tumor graphs based on expression, mutations, and interactions data. Measures based on graph-centralities are employed on resulting graphs. Ten lists of genes, eight from centrality measures and two from control measures, ordering genes with respect to their computed weights are provided as output. The final step depicted in part-c consists of analyzing the ten lists with regards to ROC, precision/recall (P/R), and GO consistencies (GOC). Two datasets, NCBI BioSystems [37] and COSMIC [38] are employed in all three analysis, whereas for the GOC analysis an additional database, the GO database [39] is also employed. Among all tested centrality-based measures *M*_*bw*_ provides the best performance in all three analyis. The *M*_*bw*_ list is further analyzed in more detail by filtering it based on a maximum weight independent set (MWIS) formulation, and the top genes from the resulting filtration go through a final literature verification step. **a** Data preparation, **b** Algorithmic computations, **c** Analysis and evaluation

We summarize the overall methodology in Fig. 1. Three main components consist of data preparation, algorithmic computations, and analysis and evaluation of results. Data preparation involves necessary preprocessing of gene expression, mutations, and network data. This is followed by the algorithmic computations step involving several graph-theoretical distance measures. The output consisting of lists ordering genes with respect to their degrees of involvement in breast cancer is evaluated in the final step. This involves ROC and precision/recall analysis as compared to two golden standard databases, COSMIC and NCBI BioSystems, and gene ontology analysis with respect to the GO database, in addition to these two golden standard datasets. The output list of the best performing measure is further filtered and a detailed review of its top genes is done through literature verfication.

### Input data sets and data preparation

We gather the breast cancer data from The Cancer Genome Atlas Project (TCGA). There are 99 instances; each instance contains data in the form of expression levels of genes in the normal and tumor tissue samples of a patient, and relevant mutation information regarding the tumor samples. For gene expression, we consider the RPKM (Reads per kilo base per million mapped reads) normalization which includes a gene length normalization of RNA-seq data and apply a threshold of 1 to assign a gene as expressed. All somatic mutations other than those marked as silent are taken into account. In addition, we employ the H. Sapiens protein-protein interaction network of the the October, 2016 version of the IntAct database [29]. The PPI network is filtered so that each interacting pair is a protein and each interaction is a physical interaction.

### Graph-theoretical framework

Let *H* be the H. Sapiens PPI network. Employing the TCGA data, for each instance *i* of the available 99 instances, we create a pair of graphs, *N*_*i*_,*T*_*i*_, corresponding to normal and tumor graphs respectively. The graph *N*_*i*_ is the subgraph of *H* induced by the node set corresponding to the set of genes expressed in the normal instance of *i*, whereas *T*_*i*_ is the subgraph induced by expressed and non-mutated genes in the tumor instance of the same sample *i*.

Let *P* be a list of pairs of graphs such that *P*=(*N*_1_,*T*_1_),…,(*N*_*r*_,*T*_*r*_), where each *N*_*i*_,*T*_*i*_ corresponds respectively to normal and tumor graphs of the instance *i*. Let $\mathcal {V} = V_{N_{1}}\cup \ldots \cup V_{N_{r}}\cup V_{T_{1}}\cup \ldots \cup V_{T_{r}}$, where *V*_*G*_ denotes the node set of a graph *G*. A measure *M*_*x*_ is a function defined on *P* that orders the nodes in $\mathcal {V}$, according to some graph-theoretical property *x*. The performance of a measure depends on how well the position of each gene in this ordering matches its revelance to the cancer under study. The measures we consider are based on the following graph-theoretical properties commonly employed in network analysis studies: *betweenness centrality, random walk distances, graph-theoretical distances, clustering coefficient, degree centrality,* and *Jaccard indices*. All of these measures are defined on the nodes of a graph. According to the traditional classification of graph-theoretical properties, the first three are *global measures*, whereas the last three are *local measures*. A global measure defined on a node is a function of the whole graph globally, whereas a local measure defined on the node usually is a function of some locality centered around the node. For the purposes of this study, we introduce a novel classification, that of *unlabeled* versus *labeled* measures. A measure of the former type on a node considers all the rest of the graph as unlabeled; the topology of the network matters but not the relationships between specific node pairs. For the latter, the node labels are important as well as the network topology. The betweenness centrality, the clustering coefficient, and the degree centrality are unlabeled measures, whereas the random walk distance, the graph-theoretical distance, and the Jaccard index based neighborhood overlaps are labeled measures. Once an ordering of the nodes with respect to a measure is determined, we apply a filtering based on *maximum weight independent sets* (MWIS) to select a subset of crucial nodes deemed important for the cancer under study.

### Unlabeled graph-theoretical measures

In what follows we provide detailed descriptions of the employed measures. For each measure we provide a node weight assignment scheme, which defines the ordering of the measure. For the following let *G*=(*V*,*E*) be an undirected graph where *V* denotes the node set and *E* denotes the edge set of the graph *G*. We first provide the definitions of four unlabeled graph-theoretical measures.

*M*_*bw*_: This measure is based on the betweenness centrality. Given *G*=(*V*,*E*), the betweenness of a node *v*∈*V* is defined as $bw_{G}(v)=\sum _{\forall s,t\in V, s\neq v\neq t}\frac {\sigma _{st}(v)}{\sigma _{st}}$ where *σ*_*st*_ is the number of shortest paths between nodes *s*,*t* and *σ*_*st*_(*v*) is the number of such paths that go through the node *v*. This value is divided by $\frac {2}{(|V|-1)(|V|-2)}$ for normalization. Note that for a node *v*∉*V*, *b* *w*_*G*_(*v*)=0 trivially. Our first measure *M*_*bw*_ sorts the nodes of $\mathcal {V}$ in non-increasing order of the node weight function *W*_*bw*_, defined for a node *v* as,

1 $$ W_{bw}(v) = \sum_{\forall (N_{i}, T_{i})\in P}\left|bw_{N_{i}}(v)-bw_{T_{i}}(v)\right|  $$

*M*_*cc*_: This measure is based on the clustering coefficient. For a node *v* in a graph *G*=(*V*,*E*) the clustering coefficient of *v*, *c* *c*_*G*_(*v*) is defined as, 2|*C*|/(*d* *e* *g*_*G*_(*v*)(*d* *e* *g*_*G*_(*v*)−1)), where *C* is the set {(*s*,*t*)∈*E*:(*v*,*s*)∈*E*,(*v*,*t*)∈*E*}. We note that for a node *v*∉*V*, *c* *c*_*G*_(*v*)=0 trivially. The measure *M*_*cc*_ sorts the nodes of $\mathcal {V}$ in non-increasing order of the weight function *W*_*cc*_, defined for a node *v* as,

2 $$ W_{cc}(v) = \sum_{\forall (N_{i}, T_{i})\in P}\left|cc_{N_{i}}(v)-cc_{T_{i}}(v)\right|  $$

*M*_*deg*1_, *M*_*deg*2_: These measures are based on the degree centrality. Let *N* *e*_*G*_(*v*) denote the set of neighbors of *v* in *G* and let $Ne_{G}^{2}(v)$ denote the set consisting of *N* *e*_*G*_(*v*) together with the neighbors of all nodes in *N* *e*_*G*_(*v*). The measure *M*_*deg*1_ sorts the nodes of $\mathcal {V}$ in non-increasing order of the node weight, defined for a node *v* as,

3 $$ W_{deg1}(v)=\sum_{\forall (N_{i}, T_{i})\in P}||Ne_{N_{i}}(v)|-|Ne_{T_{i}}(v)||  $$

whereas the measure *M*_*deg*2_ employs the weighting defined as,

4 $$ W_{deg2}(v)=\sum_{\forall (N_{i}, T_{i})\in P}\left|\left|Ne_{N_{i}}^{2}(v)\right|-\left|Ne_{T_{i}}^{2}(v)\right|\right|  $$

### Labeled graph-theoretical measures

We provide the definitions of four labeled graph-theoretical measures.

*M*_*rw*_: We employ proximity matrices based on random walks of the networks for this measure.

We note that similar methods have been employed in many previous PPI network analysis studies [30–32]. Let $Ne_{G}^+(v)=Ne_{G}(v)\cup \{v\}$. Assuming the origin of the walk is node *u*, let *P* *r* *G*′[*u*,*v*] denote the probability that the random walker is at node *v* after a certain number of time steps and *P* *r*_*G*_[*u*,*v*] denote the same probability after one more time step. Initially *P* *r* *G*′[*u*,*u*]=1, *P* *r* *G*′[*u*,*v*]=0 for *v*≠*u*. *P* *r*_*G*_[*u*,*v*] is computed from *P* *r* *G*′[*u*,*s*] for *s*∈*N* *G*+(*v*). The contribution of a neighbor *s* of *v* to *P* *r*_*G*_[*u*,*v*] is $\frac {Pr_G'[u,s]}{|Ne_{G}(s)|+1}$. A small constant *ε* is decremented from this contribution to increase the chances of the walker remaining close to the origin. Each probability is normalized by dividing it with $\sum _{v\in V}Pr_{G}[u,v]$. The procedure is repeated until the sum of the differences of probabilities with those of the previous time step does not exceed a predefined constant *threshold*. *P* *r*_*G*_[*p*,*q*]=0 trivially, if *p*∉*G* or *q*∉*G*. The measure *M*_*rw*_ based on random walk distances sorts the nodes of $\mathcal {V}$ in non-decreasing order of the node weight *W*_*rw*_, defined for a node *v* as,

5 $$ \sum_{\forall (N_{i}, T_{i})\in P}PCC\left(Pr_{N_{i}}[-,v],Pr_{T_{i}}[-,v]\right)  $$

where *P* *r*_*G*_[−,*v*] denotes the column vector corresponding to *v* in the random walks-based proximity matrix *P* *r*_*G*_ and *P* *C* *C*(*x*,*y*) denotes the Pearson correlation coefficient of the vectors *x*,*y*. *P* *r*_*G*_[*p*,*q*]=0 trivially, if *p*∉*G* or *q*∉*G*.

*M*_*gt*_: Our next measure *M*_*gt*_ is based on graph-theoretical distances and is defined in exactly the same way as the previous measure *M*_*rw*_, except now an entry *P* *r*_*G*_[*u*,*v*] of the proximity matrix *P* *r*_*G*_ defines the graph theoretical distance between nodes *u*,*v* in *G*, that is the length of the shortest path between *u*,*v*.

*M*_*j*1_, *M*_*j*2_: We define two measures based on Jaccard indices with respect to neighborhood overlaps. The measure *M*_*j*1_ sorts the nodes of $\mathcal {V}$ in non-decreasing order of the node weight, defined for a node *v* as,

6 $$ W_{j1}(v) = \sum_{\forall \left(N_{i}, T_{i}\right)\in P}{\frac{\left|Ne_{N_{i}}(v)\cap Ne_{T_{i}}(v)\right|}{\left|Ne_{N_{i}}(v)\cup Ne_{T_{i}}(v)\right|}}  $$

whereas the measure *M*_*j*2_ employs the weighting defined as,

7 $$ W_{j2}(v) = \sum_{\forall (N_{i}, T_{i})\in P}{\frac{\left|Ne_{N_{i}}^{2}(v)\cap Ne_{T_{i}}^{2}(v)\right|}{\left|Ne_{N_{i}}^{2}(v)\cup Ne_{T_{i}}^{2}(v)\right|}}  $$

### Filtering based on maximum weight independent sets

The graph-theoretical measures of the previous subsections provide a node weight assignment scheme in a way that the weight of a node represents the importance of the protein corresponding to the node regarding the cancer under study. However due to the network influence-based nature of some of these measures, they maybe susceptible to guilt by association; a node may end up with a large weight designating it a crucial protein, only because some of its neighbors have large weights. This is especially evident in measures based on betweenness centrality, random-walks, or graph-theoretical distances, as the weight of a node is dependent on the weights of its neighbors in the PPI network. In order to alleviate this issue and produce only a small set of crucial proteins, we apply a filtering on the node-weighted PPI network. The network consists of all the proteins involved in all normal, tumor instances under study and the node weights are assigned as those resulting from applying one of the mentioned graph-theoretical measures. Given a node-weighted graph *G*, the maximum weight independent set (MWIS) of *G*, is the set of nodes with maximum total weight such that no two nodes are neighbors in *G*. We note that the computational problem is NP-complete [33]. Several greedy heuristics have been investigated in [34]. The *GWMIN2* heuristic which selects the node *u* in the conflict graph $\mathcal {C}$ that maximizes $\mathcal {W}(u) / \sum _{v\in N_{\mathcal {C}}^{+}(u)}{\mathcal {W}(v)}$, where $N_{\mathcal {C}}^{+}(u)$ denotes the neighborhood of *u* in $\mathcal {C}$ together with the node *u* itself, provides better results than the rest of the known heuristics [35]. Furthermore it provides a theoretical guarantee that the weight of the output independent set is at least $\sum _{u\in V_{\mathcal {C}}} \left [{\mathcal {W}(u)}^{2} \left / \sum _{v\in N_{\mathcal {C}}^{+}(u)}{\mathcal {W}(v)}\right.\right ]$, where $V_{\mathcal {C}}$ denotes the vertex set of the conflict graph $\mathcal {C}$. Therefore the filtration step is implemented via the GWMIN2 heuristic for the MWIS problem.

## Results and discussion

We implemented the described measures in C++ using the LEDA library [36]. We show that in determining the quality of a graph-theoretical measure for identifying genes related to breast cancer, the labeled/unlabeled classification is more important than the traditional local/global classification of the measures. Furthermore we show that under this classification, the unlabeled measures perform better than the labeled measures in extracting breast cancer genes via comparison of normal/tumor network instance pairs − contrary to the intuition that the latter employs more information in the form of labeled networks. Our evaluations indicate that the measure based on betweenness centrality is the best performer in terms differential identification of breast cancer genes across all normal/tumor samples.

### Evaluations with respect to known cancer databases

Comparing against known cancer databases taken as golden standards, we measure the performances based on Receiver Operating Characteristic (ROC) and Precision/Recall (PR). As the golden standard to compare against the gene list of each of the graph-theoretical measures under study, we employ two separate databases. One is the integrated breast cancer pathway from the NCBI BioSystems database [37] and the other is the cancer Gene Census of the COSMIC database [38]. We note that whereas NCBI BioSytems data is specific to breast cancer, the COSMIC database covers genes relevant to all types of cancer. Thus we can evaluate how well each of the defined measures can identify both breast cancer-specific genes and cancer genes not specific to any certain type.

Every evaluated measure is designed so that it orders the genes from most relevant to the least. We extract the top *k* *%* genes from the list of each of the defined graph-theoretical measures, for every *k* between 1 and 100 at the increments of 1. In addition to the measures under study, we introduce two additional control measures. The first one is the *expression difference (ED)* measure which orders the genes with respect to the ED values. *E* *D*(*v*) for a gene *v* is defined as the absolute value of the difference between the number of normal and tumor samples including *v* as an expressed gene. The second control measure is the *mutation frequency (MT)* which orders the genes with respect to the number of tumor samples including them as mutated genes.

**Fig. 2:**
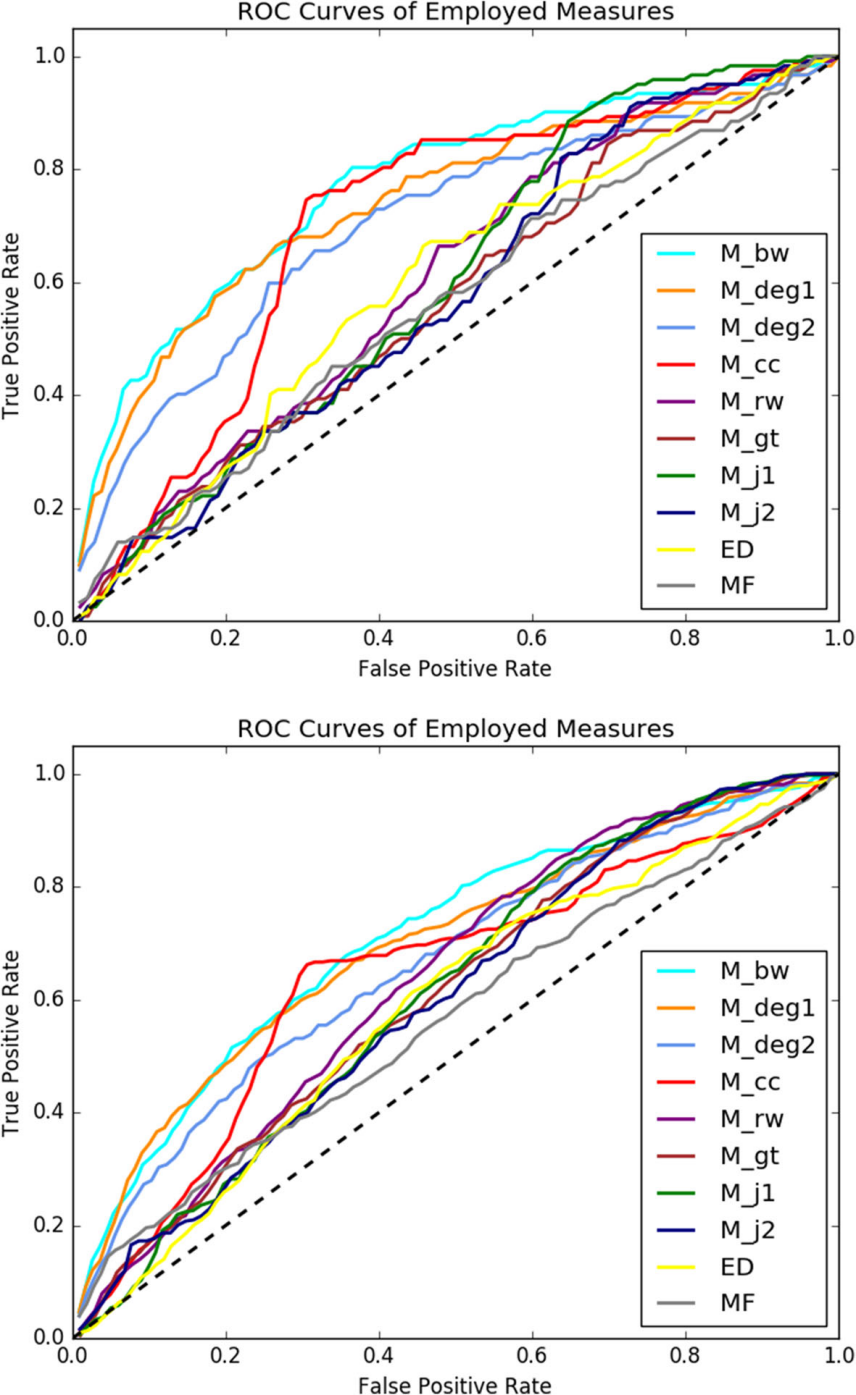
ROC Plots ROC curves for the measures under consideration for *k* changing from 1 to 100 at the increments of 1. True positive, false positive rates are with respect to the NCBI database (left) and the COSMIC database (right)

**Fig. 3:**
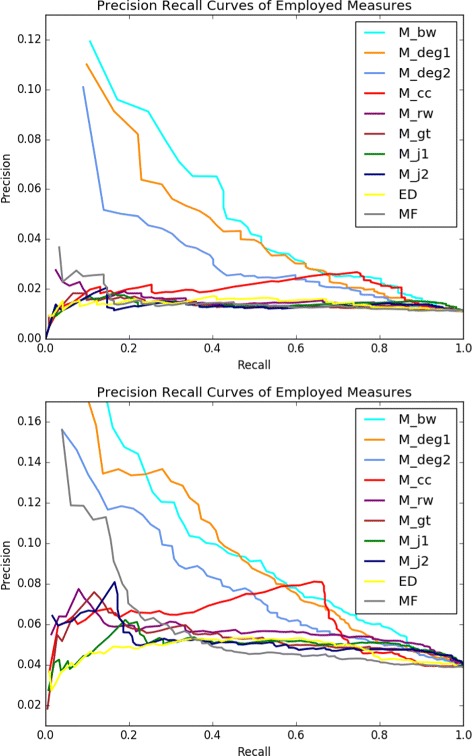
PR curves PR curves for the measures under consideration for *k* changing from 1 to 100 at the increments of 1. Precision and recall are with respect to the NCBI BioSystems database (left) and the COSMIC database (right)

Figure 2 provides the ROC curves of all the employed graph-theoretical and control measures. In the left plot, the true positives and false positives are computed based on the comparison of the top *k* *%* genes of the output list of each measure against the NCBI BioSystems database, whereas in the right plot the reference database is COSMIC. The respective PR curves are provided in Fig. 3. The corresponding AUROC and AUPR values are provided in Table 1. With respect to the ROC/PR curves and the AUROC/AUPR values the best performing measure is *M*_*bw*_. The AUROC value of the *M*_*bw*_ list as compared to the NCBI BioSystems dataset is 0.77 and its AUPR value in the same setting is 0.042. With regards to the COSMIC dataset the AUROC value of the *M*_*bw*_ list is 0.709, whereas its AUPR value is 0.091. It is clear that the rest of the unlabeled measures also perform better than the labeled measures for most values of *k*. It is interesting to note that a measure as simple as degree differentiation between normal and tumor samples across all samples, that is *M*_*deg*1_, provides a better recognition of cancer-related genes than those of the more complicated measures making use of extra information in the form of labels, such as graph-theoretical distances or Jaccard index based measures. Note also that all the unlabeled measures perform consistently better than the control measures ED and MF with respect to both of the employed golden standard cancer gene databases.

**Table 1:** AUROC and AUPR values for all the defined graph-theoretical measures and the control measures

Reference database	Measurement	*M*_*bw*_	*M*_*deg*1_	*M*_*deg*2_	*M*_*cc*_	*M*_*rw*_	*M*_*gt*_	*M*_*j*1_	*M*_*j*2_	*ED*	*MF*
NCBI Bio systems	AUROC	0.770	0.740	0.702	0.703	0.606	0.569	0.603	0.577	0.597	0.563
NCBI Bio systems	AUPR	0.042	0.037	0.027	0.020	0.015	0.013	0.014	0.013	0.014	0.014
COSMIC	AUROC	0.709	0.695	0.664	0.641	0.635	0.611	0.611	0.601	0.586	0.567
COSMIC	AUPR	0.091	0.089	0.075	0.061	0.056	0.053	0.050	0.051	0.047	0.055

The first two rows are with respect to the NCBI BioSystems database and the last two rows are with respect to the COSMIC database

### Evaluations based on gene ontology

An additional database is employed in setting up the next evaluation; the Gene Ontology (GO) database [39]. The GO database annotates proteins from several species with appropriate GO categories organized as a directed acyclic graph (DAG). In order to standardize the GO annotations of proteins, similar to the evaluation methods of [40–42], we restrict the protein annotations to level 5 of the GO DAG by ignoring the higher-level annotations and replacing the deeper-level category annotations with their ancestors at the restricted level. For a node *u*∈*V*, let *G* *O*(*u*) indicate the set of standard GO annotations of the protein corresponding to *u*. For a given list *T* of genes to be tested and a reference list *R*, we define a *GO Consistency (GOC)* score as,

$$\frac{\sum_{t\in T}\sum_{r\in R}|GO(t)\cap GO(r)| / |GO(t)\cup GO(r)|}{|R|}. $$

**Fig. 4:**
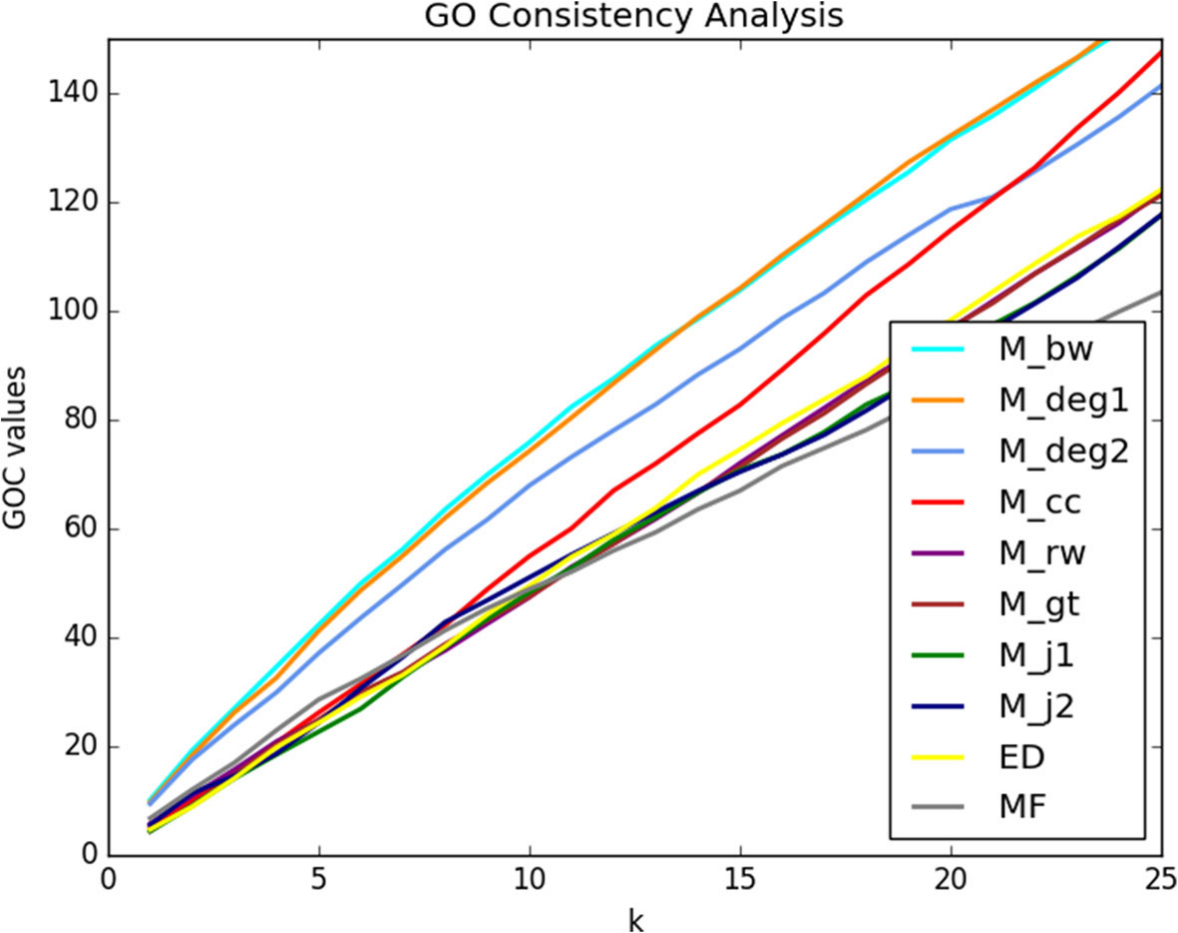
GO Consistency Evaluations The results of the GO Consistency evaluations, with regards to the NCBI BioSystems data, for *k* changing from 1 to 25 at the increments of 1

The list *T* consists of the top *k* *%* of the genes provided by one of the graph-theoretical measures under study or one of the two control measures (ED, MF), and *R* corresponds to one of the two golden standard datasets. Small values of *k* are of more interest, since the output candidate list of genes are usually intended for further detailed inspection. The results for *k* upto 25 are presented in Fig. 4. We only show the plot when the golden standard list *R* is the NCBI BioSystems pathway; the plot resulting from the GOC evaluations with respect to the COSMIC database is almost the same. It is clear that the performance trends of the evaluated measures are almost the same as those of the previous metrics based on ROC and PR, although with less emphasized differences.

Further detailed simultaneous inspection of the top two lists, *M*_*bw*_ and *M*_*deg*1_, and the GO consistency analysis with respect to the NCBI BioSystems data reveals that the top contributors to the corresponding GOC scores show significant overlap. At *k*=5, that is when the top 5% of the gene lists are considered, the four genes contributing most to the GOC score in both lists, *M*_*bw*_ and *M*_*deg*1_, are IGF1R, RAF1, YWHAB, and MYC. Note that none of these are directly listed in the golden standard gene list of the NCBI BioSystems. Among the notable GO categories they commonly or independently share with those associated with the golden standard genes are GO:0008284 (positive regulation of cell proliferation), GO:0009890 (negative regulation of biosynthetic process), GO:0016310 (phosphorylation), GO:0031325 (positive regulation of cellular metabolic process), and GO:0010648 (negative regulation of cell communication). Same analysis with respect to the COSMIC database provides CTBP2, ATF3, FHL2, NFKB2 as shared top contributors in both lists *M*_*bw*_ and *M*_*deg*1_. It is worth emphasizing that other than the last one, none of these genes is listed in the COSMIC database itself.

### Evaluations with rewired networks

Employing the criteria of the previous subsections, that is the criteria based on the ROC analysis and the GO consistency analysis with respect to the two golden standards, we further tested the two best-performing measures, *M*_*bw*_ and *M*_*deg*1_, on different networks. The networks under consideration are again based on the IntAct PPI network but modified with the introduction of varying degrees of random error via rewirings: *r* *%* of the existing edges are removed randomly and the same number of edges are inserted between random pairs of nodes not adjacent in the original network. This procedure is repeated four times giving rise to four randomly rewired networks for each value of *r*=5,10,15,20. For each rewired network the rest of the framework is the same; a pair of normal and tumor networks is generated based on the expression and mutation information of each instance by taking the induced subnetwork of the rewired network, and the relevant functions *M*_*bw*_,*M*_*deg*1_ are computed throughout all the networks. Thus, considering the induced graphs of all the samples, 99 normal and 99 tumor, in total 3168 graphs are generated and the suggested measures execute on all these graphs. The experiments on the rewired networks serve also the purpose of testing how sensitive the suggested graph-theoretical measures are to the noise in the network data.

We present the resulting AUROC and AUPR values in Table 2. Note that the true positives, false positives, precision, and recall values are computed as an average of respective values attained in four randomly rewired networks generated with the same ratio *r*. As expected the general tendency for AUROC and AUPR values with respect to both golden standard datasets is to decrease as the random rewiring ratio *r* increases. The slight discrepancies are due to the randomness in the rewirings. It should be noted that even though there is a performance decrease with growing random error in the network, this degradation in the performance is relatively small. For *M*_*bw*_, the AUROC values decrease by only 4.5*%* and 4.9*%*, respectively, for the NCBI and COSMIC databases, even with a 20% random rewiring of the original network. The respective percetages of degradation in the AUROC values of *M*_*deg*1_ are 2.2*%* and 3.3*%*. The performance degradations with respect to the AUPR values are slightly higher; for *M*_*bw*_ they are 7.1*%* and 9.9*%*, and for *M*_*deg*1_ they are 8.1*%* and 6.7*%*. This is an indication that in addition to providing good performance, the suggested measures for cancer gene prioritization are also relatively robust to random noise in the interaction network data. A closer comparative look at the rates of degradation in performances in terms of AUROC, AUPR values of *M*_*bw*_ and *M*_*deg*1_ reveals that the former gets more error-prone as the degree of noise in the network increases.

**Table 2:** AUROC and AUPR values for *M*_*bw*_ (multicolumns in the middle) and *M*_*deg*1_ (multicolumns on the right) on randomly rewired networks with rewiring ratio *r*=5*%*,10*%*,15*%*,20*%*. For a fixed ratio *r*, each value is computed as an average of four randomly rewired networks

Reference database	Measurement	0%	5%	10%	15%	20%	0%	5%	10%	15%	20%
NCBI Bio systems	AUROC	0.770	0.756	0.746	0.734	0.735	0.740	0.733	0.726	0.730	0.724
NCBI Bio systems	AUPR	0.042	0.041	0.041	0.040	0.039	0.037	0.036	0.036	0.035	0.034
COSMIC	AUROC	0.709	0.698	0.690	0.679	0.674	0.695	0.688	0.683	0.676	0.672
COSMIC	AUPR	0.091	0.088	0.086	0.082	0.082	0.089	0.087	0.086	0.085	0.083

The columns marked with 0% indicate the corresponding results for the original network. The results listed in the first two rows are with respect to the NCBI BioSystems database and those listed in the last two rows are with respect to the COSMIC database

**Fig. 5:**
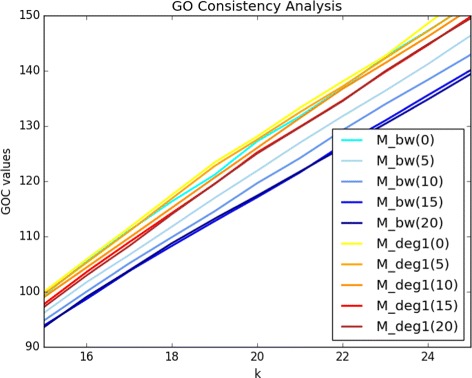
GO Consistency Evaluations on Rewired Networks The results of the GO Consistency evaluations on rewired networks, with regards to the NCBI BioSystems data, for *k* changing from 1 to 25 at the increments of 1. The plot only shows GOC values for *k*≥15, since the previous values are mostly convergent. The numbers in parantheses indicate the ratio *r*. For each ratio *r*, the experiments are run on four randomly rewired networks and an average GOC value is taken

The same phenomenon is also evident in the GO consistency analysis. The plot of GOC values of prioritized lists of *M*_*bw*_ and *M*_*deg*1_ on randomly rewired networks, for each ratio *r*, with respect to the NCBI database is provided in Fig. 5. Since the plot with respect to the COSMIC database is almost the same we do not present it. Note again that the plotted values are those averaged over the values resulting from experimental runs of four randomly rewired networks, for each *r*. As with the ROC analysis, it is clear that *M*_*bw*_ and *M*_*deg*1_ are both quite resilient to noise in the interaction network simulated via random rewirings, with *M*_*deg*1_ even more so than *M*_*bw*_.

### Comparisons against an alternative gene prioritization

We compare the results of the two measures performing the best, *M*_*bw*_,*M*_*deg*1_ against an alternative method for cancer gene prioritization. MUFFINN is similar to the gene prioritization methods suggested in this study both in terms of the employed data and the goal of disease gene prioritization in the presence of data from a limited number of patient samples [23]. In terms of input datasets, it also employs mutation data from patient samples and network data in the form of functional networks or interaction networks. The underlying hypothesis of MUFFINN is that a gene is more likely to represent a true cancer driver if it is functionally associated with other genes in an interaction network. For such a network-based mutation data analysis, they consider two ways to take into account mutational information among direct neighbors in the network. One is to consider mutations in the most frequently mutated neighbor and the second is to consider mutations in all direct neighbors with normalization by their degree connectivity. We call the former *M* *U* *F* *F* *I* *N*_*max*_ and the latter *M* *U* *F* *F* *I* *N*_*sum*_.

**Fig. 6:**
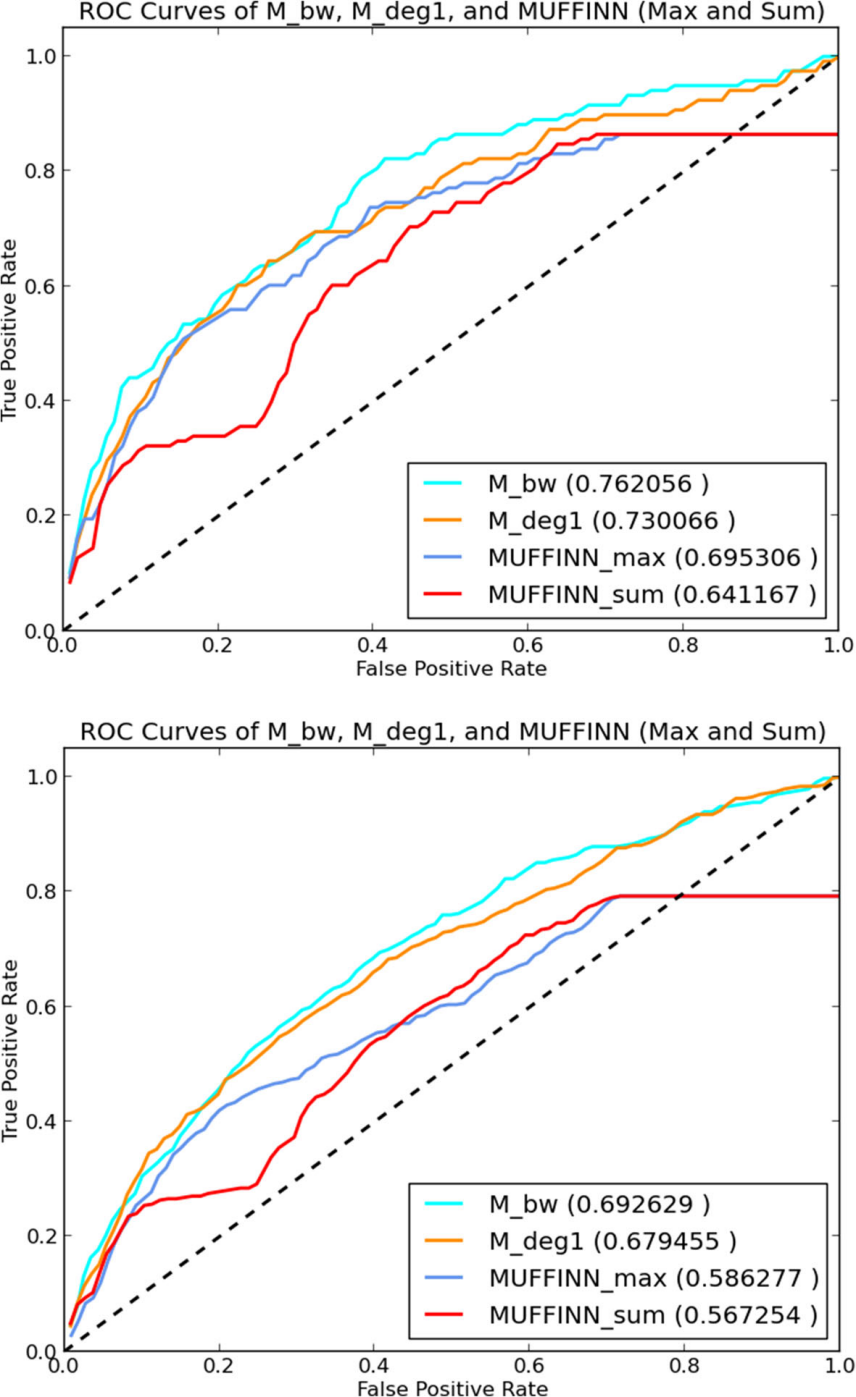
ROC Plots including MUFFINN Results ROC curves for *M*_*bw*_,*M*_*deg*1_ measures versus MUFFINN for *k* changing from 1 to 100 at the increments of 1. True positive, false positive rates are with respect to the NCBI database (left) and the COSMIC database (right)

**Fig. 7:**
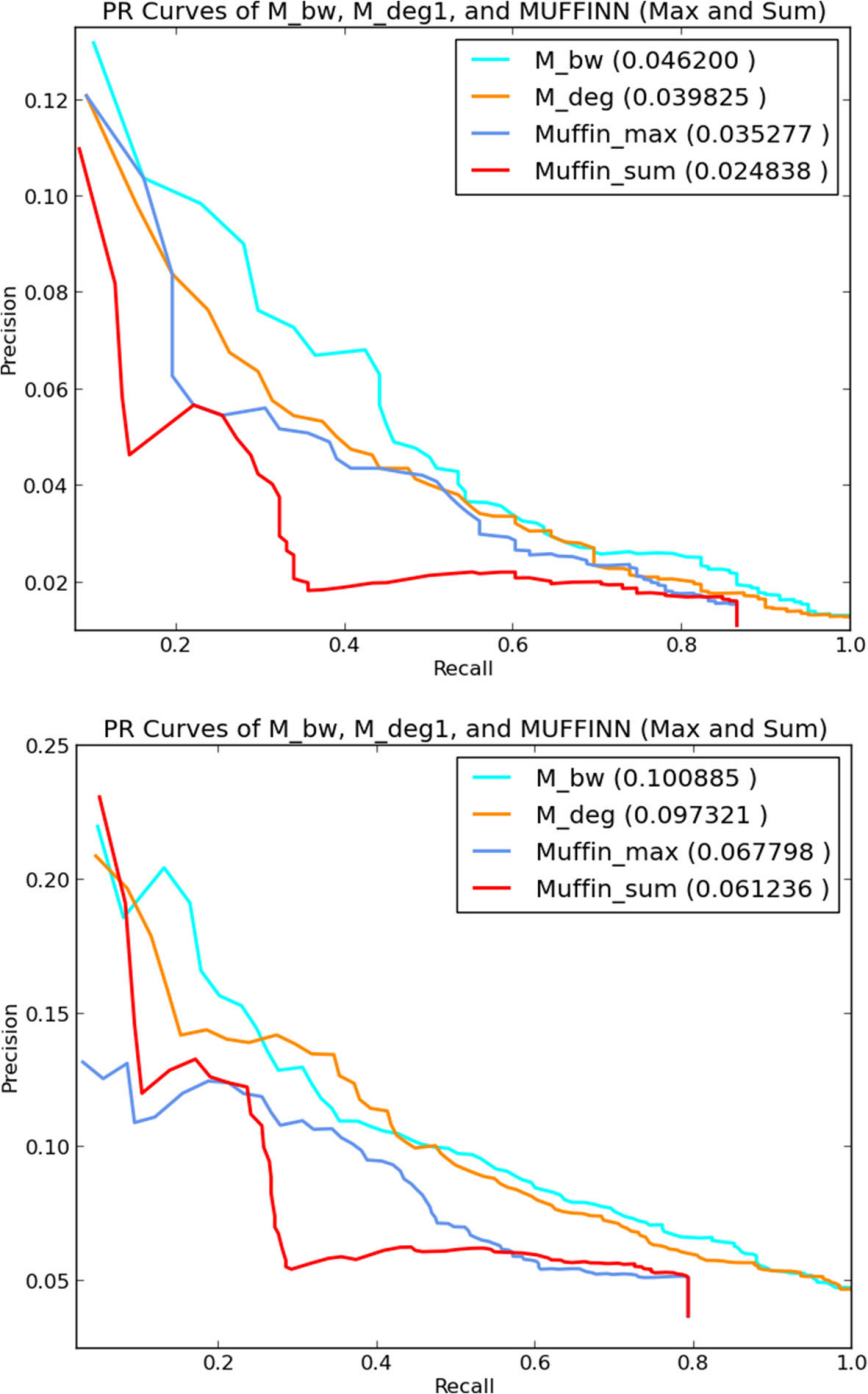
PR Plots including MUFFINN Results PR curves for the measures under consideration for *k* changing from 1 to 100 at the increments of 1. Precision and recall are with respect to the NCBI BioSystems database (left) and the COSMIC database (right)

We executed both *M* *U* *F* *F* *I* *N*_*max*_ and *M* *U* *F* *F* *I* *N*_*sum*_ with the same data employed in this study, that is the interaction network is the same IntAct network and the samples are the same TCGA samples as those used by our graph-theoretical prioritization methods. We extract the top *k* *%* genes from the list of each of the prioritization methods under comparison *M*_*bw*_,*M*_*deg*1_ and *M* *U* *F* *F* *I* *N*_*max*_, *M* *U* *F* *F* *I* *N*_*sum*_, for every *k* between 1 and 100 at the increments of 1. We then apply ROC and precision/recall analysis. In the left plot of Fig. 6 the true positives and false positives are computed based on the comparison of the top *k* *%* genes of the output list of each method against the NCBI BioSystems database, whereas in the right plot the reference database is COSMIC. The numbers in parantheses indicate the AUROC values of the relevant methods. The respective PR curves are provided in Fig. 7 and the numbers in parantheses indicate the corresponding AUPR values.

Our proposed graph-theoretical measure *M*_*bw*_ provides the largest AUROC and AUPR values with respect to both of the golden standard datasets. Even our second best measure *M*_*deg*1_ provides better results than those of both *M* *U* *F* *F* *I* *N*_*max*_ and *M* *U* *F* *F* *I* *N*_*sum*_. Note that the AUROC and AUPR values of *M*_*bw*_ and *M*_*deg*1_ are slightly different from those provided in Table 1. This is due to the fact that MUFFINN uses only genes in Concensus CDS. We filtered the reference golden standard databases to remove the rest of the genes not considered by MUFFFINN for a fair comparison, which led to slight differences in the values attained in the tests of *M*_*bw*_ and *M*_*deg*1_.

### Filtering the *M*_*bw*_ list

**Fig. 8:**
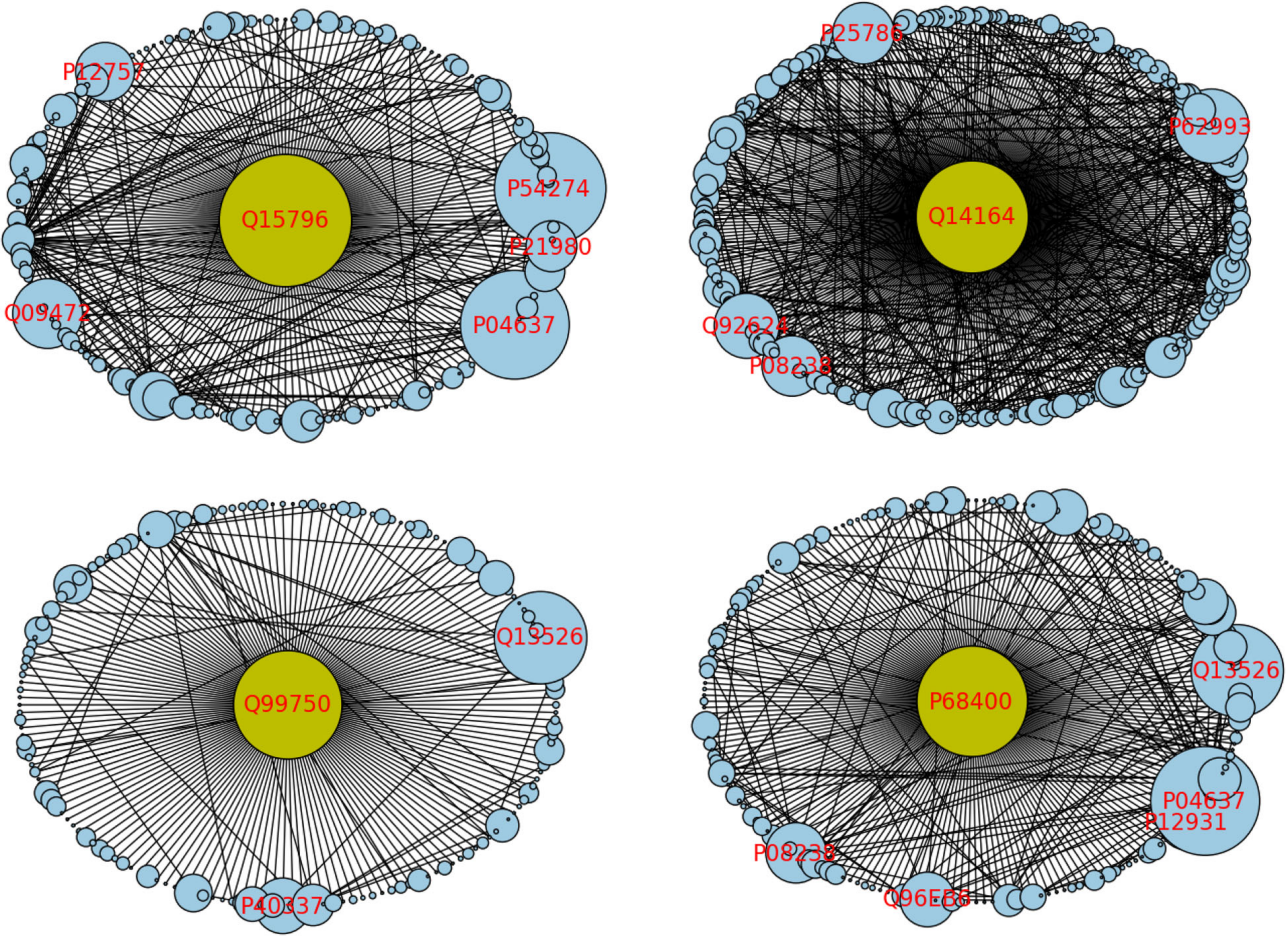
Gene Neighborhoods Top four nodes in the filtered *M*_*bw*_ list of proteins and their neighborhood subgraphs. The labeled nodes in the periphery are those in the top 50 list, but are filtered out due to the central node being selected in the MWIS

Since *M*_*bw*_ is the best performer among all the employed measures, we employ a detailed inspection of its output. The top 50 genes with respect to *M*_*bw*_ are listed in Table 3 in descending order of their weights, as shown in the *W*_*bw*_ column. We first apply the MWIS heuristic on the node-weighted PPI network to implement the filtration. The rows of Table 3 that are marked with bold correspond to filtered nodes, that is they are in the MWIS output. The column marked with *N* provides the number of normal samples including the gene as an expressed gene, the column marked with *T* provides the corresponding number for tumor samples, the column marked with *M* provides the number of tumor samples the gene occurs as mutated, the column marked with *G* *S*_1_ indicates whether the gene is listed in the first golden standard dataset, NCBI BioSystems, the column marked with *G* *S*_2_ provides the analogous information regarding the COSMIC database, and finally the last column provides the list of genes presented in the table that are in the MWIS of the *W*_*bw*_-weighted PPI network and that are neighbors of the given gene in the network. As a sample Fig. 8 provides the neighborhood subgraphs of the top four MWIS genes of the list. Each subgraph is induced by the protein corresponding to the center node and its neighbors in the PPI network. Nodes are weighted with corresponding *W*_*bw*_ values. The labeled nodes in the periphery are those in the top 50 list, but are filtered out from MWIS since the central node is included in MWIS.

**Table 3:** Top 50 genes with respect to *M*_*bw*_

	Id	Gene	*W*_*bw*_	*N*	*T*	*M*	*G* *S*_1_	*G* *S*_2_	Top neighbors in MWIS
**1**	**Q15796**	**SMAD2**	**0.896534**	**64**	**31**	**0**	**Yes**	**Yes**	
**2**	**Q14164**	**IKBKE**	**0.646178**	**1**	**16**	**0**	**No**	**No**	
3	P54274	TERF1	0.638236	59	55	0	No	No	1, 20, 34
**4**	**P68400**	**CSNK2A1**	**0.626417**	**85**	**91**	**0**	**No**	**No**	
5	P04637	TP53	0.60796	98	97	17	Yes	Yes	1, 4, 7, 8, 34, 42, 44
**6**	**Q99750**	**MDFI**	**0.60268**	**60**	**36**	**0**	**No**	**No**	
**7**	**P04183**	**TK1**	**0.578819**	**10**	**92**	**0**	**No**	**No**	
**8**	**Q96SB4**	**SRPK1**	**0.457947**	**0**	**36**	**0**	**No**	**No**	
9	Q13526	PIN1	0.438312	68	77	0	No	No	4, 6, 46
10	P46108	CRK	0.297179	96	70	0	No	No	21, 25, 35
11	P62993	GRB2	0.284621	97	98	0	No	No	2, 46
**12**	**Q99558**	**MAP3K14**	**0.283214**	**45**	**17**	**0**	**No**	**No**	
13	Q9Y6K9	IKBKG	0.271303	4	10	0	No	No	12, 19
**14**	**Q13387**	**MAPK8IP2**	**0.269689**	**1**	**60**	**0**	**No**	**No**	
**15**	**Q13233**	**MAP3K1**	**0.255928**	**68**	**63**	**4**	**No**	**Yes**	
16	Q09472	EP300	0.250913	94	89	0	Yes	Yes	1, 34, 35
**17**	**P21246**	**PTN**	**0.248842**	**53**	**2**	**0**	**No**	**No**	
**18**	**P20333**	**TNFRSF1B**	**0.234258**	**71**	**31**	**0**	**No**	**No**	
**19**	**Q99759**	**MAP3K3**	**0.233384**	**19**	**4**	**0**	**No**	**No**	
**20**	**O60341**	**KDM1A**	**0.229576**	**79**	**93**	**0**	**No**	**No**	
**21**	**Q92569**	**PIK3R3**	**0.225153**	**29**	**76**	**0**	**No**	**No**	
22	Q15714	TSC22D1	0.22131	99	76	0	No	No	17, 42
23	Q92624	APPBP2	0.217109	41	38	0	No	No	2
24	Q15047	SETDB1	0.212244	81	95	0	No	No	7, 20
**25**	**P30480**	**HLA-B**	**0.20772**	**99**	**99**	**0**	**No**	**No**	
26	P25791	LMO2	0.197168	38	2	0	No	Yes	
27	P25786	PSMA1	0.194226	4	36	0	No	No	2, 25, 40
28	P08238	HSP90AB1	0.187144	99	99	0	No	No	2, 4, 8, 12, 15, 19, 34, 42, 49
**29**	**P14921**	**ETS1**	**0.184287**	**82**	**42**	**0**	**No**	**No**	
30	P12757	SKIL	0.177551	32	73	1	No	No	1
31	P03372	ESR1	0.175797	4	36	0	Yes	Yes	15
32	Q16539	MAPK14	0.173795	60	33	0	No	No	8
33	P63104	YWHAZ	0.172695	99	99	0	No	No	7, 19, 42
**34**	**P22736**	**NR4A1**	**0.170823**	**84**	**44**	**0**	**No**	**No**	
**35**	**O15162**	**PLSCR1**	**0.169279**	**90**	**76**	**0**	**No**	**No**	
36	P12931	SRC	0.169016	4	28	0	No	Yes	4, 21
37	P04626	ERBB2	0.164884	88	98	1	No	Yes	8, 14, 21
38	P40337	VHL	0.158704	99	99	0	No	Yes	6
39	Q96EB6	SIRT1	0.153643	94	68	1	Yes	No	4
**40**	**P40692**	**MLH1**	**0.147726**	**1**	**20**	**0**	**No**	**Yes**	
41	Q9Y4K3	TRAF6	0.145986	2	2	0	No	No	12, 19
**42**	**P04792**	**HSPB1**	**0.14386**	**99**	**99**	**0**	**No**	**No**	
43	Q5UIP0	RIF1	0.139848	26	20	1	No	No	7, 42
**44**	**Q96PM5**	**RCHY1**	**0.136242**	**42**	**22**	**1**	**No**	**No**	
**45**	**Q92754**	**TFAP2C**	**0.135754**	**86**	**98**	**1**	**No**	**No**	
**46**	**Q9Y478**	**PRKAB1**	**0.134543**	**98**	**96**	**0**	**No**	**No**	
47	O14920	IKBKB	0.132882	62	53	0	No	Yes	12, 15, 19, 45
48	P21980	TGM2	0.132793	60	81	0	No	No	1
**49**	**Q9Y572**	**RIPK3**	**0.13256**	**92**	**75**	**0**	**No**	**No**	
50	P42858	HTT	0.131761	4	10	0	No	No	21

The first column provides the Uniprot id of the gene, the second column provides the gene name. The third column provides the weight of each gene based on *M*_*bw*_. The fourth and the fifth columns provide the number of instances each gene is expressed in the normal and tumor samples respectively. The sixth column provides the number of mutations of a gene observed throughout all the tumor samples in the dataset. The seventh column indicates whether the gene is listed in the breast cancer pathway of the first golden standard, the NCBI BioSystems, whereas the eight columnd indicates whether it is listed in the second golden standard, the COSMIC database. The last column provides the set of PPI network neighbors of the corresponding gene from the top 50 list that are also in MWIS

A literature review of the proteins resulting from filtration that are marked in bold in the table reveals that almost all of them play significant roles in breast cancer. We provide a review of each such protein not verified by either of the employed golden standard datasets. IKBKE has been shown to be a breast cancer oncogene via integrative genomic approaches [43]. More recently, Sang Bae et al. have shown that CK2/CSNK2A1 phosphorylates SIRT6 and is involved in the progression of breast carcinoma [44]. MDFI is considered a candidate tumor suppressor gene involved in cellular and viral transcriptional regulation [45]. TK1 is a widely accepted biomarker for cancer [46]. Roosmalen et al. have suggested SRPK1 as a breast cancer metastasis determinant via tumor cell migration screen [47]. The relationship between MAP3K1 and breast cancer detailing the possible mechanisms MAP3K1 mutations affect pathways important in breast carcinoma has been discussed in [48]. The role of PTN in the malignant progression of breast cancer is well established since early work [49]. The role of TNFRSF1B in triple-negative breast cancer (TNBC) has been studied in [50]. It is suggested that MAP3K3 contributes to breast carcinogenesis and MAP3K3 may prove to be a valuable therapeutic target in patients MAP3K3-amplified breast cancers [51]. KDM1A/LSD1 is suggested as a predictive marker for breast carcinogenesis and a novel attractive therapeutic target for treatment of ER-negative breast cancers. PIK3R3 is identified as one of the crucial genes for regulating triple negative breast cancer cell migration [52]. It is shown that HLA class I expression, including HLA-B, in breast cancer was significantly associated with nodal metastasis, TNM, lymphatic invasion, and venous invasion [53]. Furlan et al. have shown, in vitro and in vivo, an unsuspected facet of ETS1 in breast tumorigenesis. They show that while promoting malignancy through the acquisition of invasive features, ETS1 also attenuates breast tumor cell growth and could therefore repress the growth of primary tumors and metastases [54]. Due to the NR4A1-dependent regulation of *T* *G* *F* *β* signaling, NR4A1 is considered to promote breast cancer invasion and metastasis [55]. It is shown that PLSCR1 binds to onzin, a negative transcriptional regulatory target of c-Myc regulating cell proliferation which potentially implicates the role of PLSCR1 in cancer cell survival and proliferation [56]. HSPB1 downregulation in human breast cancer cells has been shown to induce upregulation of PTEN, a tumor suppressor gene [57]. Human Pirh2 (p53-induced RING-H2 protein) is encoded by the RCHY1 gene. Decrease of Pirh2 expression in the breast cancer cells result in reduced tumor cell growth via the inhibition of cell proliferation and the interruption of cell cycle transition [58]. It is suggested that TFAP2C overexpression correlates with poor overall survival after 10 years of diagnosis of breast cancer [59]. Koo et al. have proposed that RIPK3 deficiency is positively selected during tumor growth/development in breast cancer [60].

In addition to these genes already verified by relevant literature, the MWIS genes in the top 50 list contains three novel genes with indefinite associations to breast cancer: MAP3K14, MAPK8IP2, and PRKAB1. Although not verified by literature, the *M*_*bw*_ measure suggests these three as candidate breast cancer genes that deserve further investigation.

## Conclusion

We defined a framework to evaluate the performances of several network measures in differentially identifying cancer-related genes on tumor versus normal network instance pairs. We applied this framework on the breast cancer data. Two separate classifications of the network measures are defined; local/global and labeled/unlabeled. We demonstrate that on the available data, the local/global classification is not as reliable a source for separating the good performing measures from bad ones as the labeled/unlabeled classification. Unlabeled network measures surprisingly outperform labeled ones. The best performing measure is based on betweenness centrality, a global and unlabeled network measure. Applying the measures employed in this study to instances from various other types of cancer is part of the planned future work. Extending the defined measures to node-weighted, edge-weighted graphs, where a node weight represents the expression level of the corresponding gene and the edge weight represents the confidence attributed to the corresponding interaction in the PPI network may also provide valuable information in terms of cancer-related genes identification. We finally note that the main purpose of MWIS filtration is to compress the list of all scored genes into a shorter list of genes, for detailed inspection, such as in the form of literature verification as is done in this study. Although such a compression is not done blindly, by simply taking the top 50 genes for instance, and the effects of guilt-by-association are taken into consideration through the heuristic idea of independent sets for providing true positives, the compressed list can be susceptible to error in terms of false negatives. Due to the nature of independent sets, at most one of the two possibly high scoring genes is provided for every interacting pair. Thus further biological evaluations could focus on such high scoring pairs with one gene present, the other absent in the compressed list, and the significant genes in gene neighborhoods as in Fig. 8 for further simultaneous inspections.

## References

[1] Sager R (1997). Expression genetics in cancer: Shifting the focus from dna to rna. Proc Natl Acad Sci.

[2] Dimitrakopoulos CM, Beerenwinkel N. Computational approaches for the identification of cancer genes and pathways. Wiley Interdiscip Rev Syst Biol Med. 2017;9(1). 10.1002/wsbm.1364 PMC5215607 27863091

[3] Leiserson MDM, Blokh D, Sharan R, Raphael BJ (2013). Simultaneous identification of multiple driver pathways in cancer. PLOS Comput Biol.

[4] Miller CA, Settle SH, Sulman EP, Aldape KD, Milosavljevic A (2011). Discovering functional modules by identifying recurrent and mutually exclusive mutational patterns in tumors. BMC Med Genet.

[5] Vandin F, Upfal E, Raphael BJ (2011). Algorithms for detecting significantly mutated pathways in cancer. J Comput Biol J Comput Mol Cell Biol.

[6] Vandin F, Upfal E, Raphael BJ (2012). De novo discovery of mutated driver pathways in cancer. Genome Res.

[7] Babur O, Gönen M, Aksoy BAA, Schultz N, Ciriello G, Sander C, Demir E. Systematic identification of cancer driving signaling pathways based on mutual exclusivity of genomic alterations. Genome Biol.2015;16. 10.1186/s13059-015-0612-6 PMC4381444 25887147

[8] Lee Y, Hwang S, Kim J, Park T, Kim Y, Myeong H, Kwon K, Jang C, Noh Y, Kim S (2015). Topological network analysis of differentially expressed genes in cancer cells with acquired gefitinib resistance. Cancer Genomics Proteomics.

[9] Liang P, Pardee AB (2003). Eanalysing differential gene expression in cancer. Nat Rev Cancer.

[10] Grützmann R, Boriss H, Ammerpohl O, Lüttges J, Kalthoff H, Schackert HK, Klöppel G, Saeger HD, Pilarsky C (2005). Meta-analysis of microarray data on pancreatic cancer defines a set of commonly dysregulated genes. Oncogene.

[11] Ping Y, Deng Y, Wang L, Zhang H, Zhang Y, Xu C, Zhao H, Fan H, Yu F, Xiao Y, Li X (2015). Identifying core gene modules in glioblastoma based on multilayer factor-mediated dysfunctional regulatory networks through integrating multi-dimensional genomic data. Nucleic Acids Res.

[12] Network TCGAR (2013). Comprehensive molecular characterization of clear cell renal cell carcinoma. Nature.

[13] Ruffalo M, Koyutürk M, Sharan R (2015). Network-Based Integration of Disparate Omic Data To Identify “Silent Players” in Cancer. PLoS Comput Biol.

[14] Porta-Pardo E, Garcia-Alonso L, Hrabe T, Dopazo J, Godzik A (2015). A pan-cancer catalogue of cancer driver protein interaction interfaces. PLOS Comput Biol.

[15] Chen X, Yan C, Zhang X, You Z. Long non-coding rnas and complex diseases: from experimental results to computational models. Brieings in Bioinformatics. 2016;:1–19. doi:10.1093/bib/bbw060. 10.1093/bib/bbw060 PMC5862301 27345524

[16] Chen X, You ZH, Yan GY, Gong DW (2016). Irwrlda: improved random walk with restart for lncrna-disease association prediction. Oncotarget.

[17] You ZH, Huang ZA, Zhu Z, Yan GY, Li ZW, Wen Z, Chen X (2017). Pbmda: A novel and effective path-based computational model for mirna-disease association prediction. PLOS Comput Biol.

[18] Chen X, Yan C, Zhang X, You Z, Huang Y, Yan G (2016). Hgimda: Heterogeneous graph inference for mirna-disease association prediction. Oncotarget.

[19] Chen X. Katzlda: Katz measure for the lncrna-disease association prediction. Sci Rep.2015;5. 10.1038/srep16840 PMC4649494 26577439

[20] Garcia-Alonso L, Jiménez-Almazán J, Carbonell-Caballero J, Vela-Boza A, Santoyo-López J, Antiñolo G, Dopazo J. The role of the interactome in the maintenance of deleterious variability in human populations. Mol Syst Biol. 2014;10(9). 10.15252/msb.20145222 PMC4299661 25261458

[21] Leiserson MD, Vandin F, Wu H-TT, Dobson JR, Eldridge JV, Thomas JL, Papoutsaki A, Kim Y, Niu B, McLellan M, Lawrence MS, Gonzalez-Perez A, Tamborero D, Cheng Y, Ryslik GA, Lopez-Bigas N, Getz G, Ding L, Raphael BJ (2015). Pan-cancer network analysis identifies combinations of rare somatic mutations across pathways and protein complexes. Nat Genet.

[22] Kim YA, Cho DY, Dao P, Przytycka TM (2015). Memcover: integrated analysis of mutual exclusivity and functional network reveals dysregulated pathways across multiple cancer types. Bioinformatics.

[23] Cho A, Shim JE, Kim E, Supek F, Lehner B, Lee I (2016). Muffinn: cancer gene discovery via network analysis of somatic mutation data. Genome Biol.

[24] Gross AM, Kreisberg JF, Ideker T (2015). Analysis of matched tumor and normal profiles reveals common transcriptional and epigenetic signals shared across cancer types. PLoS One.

[25] Costa GD, Gomig T, Kaviski R, Sousa KS, Kukolj C, Lima RD, Urban CDA, Cavalli I, Ribeiro E (2015). Comparative proteomics of tumor and paired normal breast tissue highlights potential biomarkers in breast cancer. Cancer Genomics Proteomics.

[26] Gaude E, Frezza C (2012). Tissue-specific and convergent metabolic transformation of cancer correlates with metastatic potential and patient survival. Nat Commun.

[27] Wang J, Chen G, Li M, Pan Y (2011). Integration of breast cancer gene signatures based on graph centrality. BMC Syst Biol.

[28] Ramadan E, Alinsaif S, Hassan MR (2016). Network topology measures for identifying disease-gene association in breast cancer. BMC Bioinformatics.

[29] Orchard S, Ammari M, Aranda B, Breuza L, Briganti L, Broackes-Carter F, Campbell NH, Chavali G, Chen C, del-Toro N, Duesbury M, Dumousseau M, Galeota E, Hinz U, Iannuccelli M, Jagannathan S, Jimenez R, Khadake J, Lagreid A, Licata L, Lovering RC, Meldal B, Melidoni AN, Milagros M, Peluso D, Perfetto L, Porras P, Raghunath A, Ricard-Blum S, Roechert B, Stutz A, Tognolli M, van Roey K, Cesareni G, Hermjakob H (2014). The mintact project–intact as a common curation platform for 11 molecular interaction databases. Nucleic Acids Res.

[30] Cao M, Pietras CM, Feng X, Doroschak KJ, Schaffner T, Park J, Zhang H, Cowen LJ, Hescott BJ (2014). New directions for diffusion-based network prediction of protein function: incorporating pathways with confidence. Bioinformatics.

[31] Wang Y, Qian X (2014). Functional module identification in protein interaction networks by interaction patterns. Bioinformatics.

[32] Leiserson MD, Vandin F, Wu H-TT, Dobson JR, Eldridge JV, Thomas JL, Papoutsaki A, Kim Y, Niu B, McLellan M, Lawrence MS, Gonzalez-Perez A, Tamborero D, Cheng Y, Ryslik GA, Lopez-Bigas N, Getz G, Ding L, Raphael BJ (2015). Pan-cancer network analysis identifies combinations of rare somatic mutations across pathways and protein complexes. Nat Genet.

[33] Garey MR, Johnson DS (1979). Computers and Intractability: A Guide to the Theory of NP-Completeness..

[34] Sakai S, Togasaki M, Yamazaki K (2003). A note on greedy algorithms for the maximum weighted independent set problem. Discrete Appl Math.

[35] Abaka G, Biyikoglu T, Erten C (2013). Campways: constrained alignment framework for the comparative analysis of a pair of metabolic pathways. Bioinformatics.

[36] Mehlhorn K, Naher S (1999). Leda: A Platform for Combinatorial and Geometric Computing..

[37] Geer LY, Marchler-Bauer A, Geer RC, Han L, He J, He S, Liu C, Shi W, Bryant SH (2010). The NCBI biosystems database. Nucleic Acids Res.

[38] Forbes SA, Beare D, Boutselakis H, Bamford S, Bindal N, Tate J, Cole CG, Ward S, Dawson E, Ponting L, Stefancsik R, Harsha B, Kok CY, Jia M, Jubb H, Sondka Z, Thompson S, De T, Campbell PJ (2017). Cosmic: somatic cancer genetics at high-resolution. Nucleic Acids Res.

[39] Ashburner M, Ball CA, Blake JA (2000). Gene Ontology: tool for the unification of biology. Nat Genet.

[40] Singh R, Xu J, Berger B (2008). Global alignment of multiple protein interaction networks with application to functional orthology detection. Proceedings of the National Academy of Sciences.

[41] Liao CS, Lu K, Baym M, Singh R, Berger B (2009). Isorankn: spectral methods for global alignment of multiple protein networks. Bioinformatics.

[42] Aladağ AE, Erten C (2013). Spinal: Scalable protein interaction network alignment. Bioinformatics.

[43] Boehm JS, Zhao JJ, Yao J, Kim SY, Firestein R, Dunn IF, Sjostrom SK, Garraway LA, Weremowicz S, Richardson AL, Greulich H, Stewart CJ, Mulvey LA, Shen RR, Ambrogio L, Hirozane-Kishikawa T, Hill DE, Vidal M, Meyerson M, Grenier JK, Hinkle G, Root DE, Roberts TM, Lander ES, Polyak K, Hahn WC (2007). Integrative genomic approaches identify {IKBKE} as a breast cancer oncogene. Cell.

[44] Bae JS, Park SH, Jamiyandorj U, Kim KM, Noh SJ, Kim JR, Park HJ, Kwon KS, Jung SH, Park HS, Park BH, Lee H, Moon WS, Sylvester KG, Jang KY (2016). Ck2 *α*/csnk2a1 phosphorylates sirt6 and is involved in the progression of breast carcinoma and predicts shorter survival of diagnosed patients. Am J Pathol.

[45] Kusano S, Yoshimitsu M, Hachiman M, Ikeda M (2015). I-mfa domain proteins specifically interact with htlv-1 tax and repress its transactivating functions. Virology.

[46] Alegre M, Robison R, O’Neill K (2013). Thymidine kinase 1: A universal marker for cancer. Cancer Clin Oncol.

[47] van Roosmalen W, Le Dévédec SE, Golani O, Smid M, Pulyakhina I, Timmermans AM, Look MP, Zi D, Pont C, de Graauw M, Naffar-Abu-Amara S, Kirsanova C, Rustici G, Hoen PA, Martens JWM, Foekens JA, Geiger B, van de Water B (2015). Tumor cell migration screen identifies SRPK1 as breast cancer metastasis determinant. J Clin Investig.

[48] Ellis M, Ding L, Shen D, Luo J, Suman V, Wallis J, Van Tine B, Hoog J, Goiffon R, Goldstein T, Ng S, Lin L, Crowder R, Snider J, Ballman K, Weber J, Chen K, Koboldt D, Kandoth C, Schierding W, McMichael J, Miller C, Lu C, Harris C, McLellan M, Wendl M, Deschryver K, Allred D, Esserman L, Unzeitig G, Margenthaler J, Babiera G, Marcom P, Guenther J, Leitch M, Hunt K, Olson J, Tao Y, Maher C, Fulton L, Fulton R, Harrison M, Oberkfell B, Du F, Demeter R, Vickery T, Elhammali A, Piwnica-Worms H, McDonald S, Watson M, Dooling D, Ota D, Chang L, Bose R, Ley T, Piwnica-Worms D, Stuart J, Wilson R, Mardis E (2012). Whole-genome analysis informs breast cancer response to aromatase inhibition. Nature.

[49] Tate Riegel A, Wellstein A (1994). The potential role of the heparin-binding growth factor pleiotrophin in breast cancer. Breast Cancer Res Treat.

[50] Li HH, Zhu H, Liu LS, Huang Y, Guo J, Li J, Sun XP, Chang CX, Wang ZH, Zhaia K (2015). Tumour necrosis factor- *α* gene polymorphism is associated with metastasis in patients with triple negative breast cancer. Sci Rep.

[51] Zhang H, Fan Y, Ge N, Wang X, Sun W, Mao R, Bu W, Creighton C, Zheng P, Vasudevan S, An L, Yang J, Zhao Y, Zhang H, Li X, Rao P, Leung E, Lu Y, Gray J, Schiff R, Hilsenbeck S, Osborne C, Yang J (2014). Amplification and over-expression of map3k3 gene in human breast cancer promotes formation and survival of breast cancer cells. J Pathol.

[52] Klahan S, Wu M, Hsi E, Huang C, Hou M, Chang W. Computational analysis of mrna expression profiles identifies the itg family and pik3r3 as crucial genes for regulating triple negative breast cancer cell migration. BioMed Res Int. 2014;2014. doi:10.1155/2014/536591. 10.1155/2014/536591 PMC4032728 24982892

[53] Kaneko K, IshigamiEmail S, Kijima Y, Funasako Y, Hirata M, Okumura H, Shinchi H, Koriyama C, Ueno S, Yoshinaka H, Natsugoe S (2011). Clinical implication of hla class i expression in breast cancer. Cancer Clin Oncol.

[54] Furlan A, Vercamer C, Bouali F, Damour I, Chotteau-Lelievre A, Wernert N, Desbiens X, Pourtier A (2014). Ets-1 controls breast cancer cell balance between invasion and growth. Int J Cancer.

[55] Zhou F, Drabsch Y, Dekker TJ, De Vinuesa AG, Li Y, Hawinkels LJ, Sheppard KA, Goumans MJ, Luwor RB, De Vries CJ, et al.Nuclear receptor nr4a1 promotes breast cancer invasion and metastasis by activating tgf- *β* signalling. Nat Commun.2014;5. 10.1038/ncomms4388 24584437

[56] Kodigepalli KM, Bowers K, Sharp A, Nanjundan M (2015). Roles and regulation of phospholipid scramblases. FEBS Lett.

[57] Cayado-Gutiérrez N, Moncalero VL, Rosales EM, Berón W, Salvatierra EE, Alvarez-Olmedo D, Radrizzani M, Ciocca DR (2013). Downregulation of hsp27 (hspb1) in mcf-7 human breast cancer cells induces upregulation of pten. Cell Stress Chaperones.

[58] Yang S, Chen Y, Sun F, Ni Q, Wang H, Huang Y, Zhang C, Liu K, Wang S, Qiu J (2016). Downregulated pirh2 can decrease the proliferation of breast cancer cells. Arch Med Res.

[59] Perkins SM, Bales C, Vladislav T, Althouse S, Miller KD, Sandusky G, Badve S, Nakshatri H (2015). Tfap2c expression in breast cancer: correlation with overall survival beyond 10 years of initial diagnosis. Breast Cancer Res Treat.

[60] Koo GB, Morgan MJ, Lee DG, Kim WJ, Yoon JH, Koo JS, Kim SI, Kim SJ, Son MK, Hong SS (2015). Methylation-dependent loss of rip3 expression in cancer represses programmed necrosis in response to chemotherapeutics. Cell Res.

